# MovingU: A prospective cohort study to understand behavioural and environmental contexts influencing physical activity during the transition into emerging adulthood

**DOI:** 10.1186/s12889-016-3372-7

**Published:** 2016-08-05

**Authors:** Matthew Y. W. Kwan, Chloe Bedard, Sara King-Dowling, Sarah Wellman, John Cairney

**Affiliations:** 1 Department of Family Medicine, McMaster University, 100 Main Street, DBHSC 5th Floor, Hamilton, ON L8P 1H6 Canada; 2 Michael G. DeGroote School of Medicine Niagara Regional Campus, McMaster University, 500 Glenridge Avenue, St., Catharines, ON L2S 3A1 Canada; 3 Department of Clinical Epidemiology and Biostatistics, McMaster University, 1280 Main Street West, Hamilton, ON L8S 4L8 Canada; 4 Department of Kinesiology, McMaster University, 1280 Main Street West, Hamilton, ON L8S 4L8 Canada

**Keywords:** Emerging adulthood, Physical activity, Physical activity determinants, Accelerometers, Ecological momentary assessment, Social cognitive theory

## Abstract

**Background:**

Children and youth are often considered the most active segment of the population, however, research indicates that physical activity (PA) tends to peak during the adolescent years, declining thereafter with age. In particular, the acute transition out of high school is a period for which individuals appear to be at high-risk for becoming less active. Relatively few studies have investigated the factors influencing the changes in PA during this transition period. Therefore the purpose of the MovingU study is to gain a comprehensive understanding of the behavioural patterns and the socio-ecological factors related to the changes in PA during the transition out of high school.

**Methods/Design:**

MovingU is comprised of two phases. Phase I is a prospective cohort design and aims to follow 120 students in their last year of high school through to their first year out of high school. Students will be asked to complete questionnaires measuring various psychosocial and socio-environmental variables (e.g., self-efficacy and distress) four times throughout this transition period. Students will also be given a wrist-worn accelerometer to wear for 7-days at each of the four assessments. Phase II is a cross-sectional study involving 100 first-year university students. Students will be asked to complete the same questionnaire from phase I, wear a wrist-worn accelerometer for 5-days, and complete ecological momentary assessments (EMA) using their smartphones at randomly selected times throughout the day for 5-days. EMA items will capture information regarding contextual and momentary correlates of PA.

**Discussion:**

The MovingU study represents the first to evaluate the social and environmental influences of PA behaviour changes, including the use of intensive real-time data capture strategies during the transition out of high school. This information will be critical in the development of interventions aimed to prevent or attenuate such drastic declines in PA during emerging adulthood period.

## Background

Alarmingly high rates of physical inactivity are a major public health concern [1–3], carrying with it significant social and financial burden. In Canada alone, inactivity is attributed to an estimated annual economic cost of $5.6 billion [4, 5]. While most research has focused on increasing PA participation, little work has been aimed at preventing PA declines. For young people, PA includes not only habitual (e.g., active transport) and intentional (e.g., exercise) forms of activity, but also participation in organized sport. Children and youth are often considered the most active segment of the population, however, research indicates that PA tends to peak during the adolescent years, declining thereafter with age [6–8]. Consequently, this has led to calls for more research to focus on declining participation in PA; and in particular, during the acute transition out of high school – a period for which individuals appear to be at high-risk for becoming less active [9–13].

Irrespective of the various self-reported measures of PA used to study changes in PA behaviours, there is compelling evidence showing individuals engaging in much more leisure-time PA while at high school compared to the period following high school graduation (i.e., young adults’ transition into postsecondary or the workforce). Specifically, studies have found significant decreases in both moderate-to-vigorous physical activity (MVPA); [9, 11, 13, 14] and in overall estimated energy expenditure based on detailed self-reported information regarding specific activity engagement [10, 15].

Despite the consistency in the narrative, there are two notable limitations in the extant PA literature focused on the emerging adulthood period. First, our knowledge is largely based on self-reported PA measures [9–13]. Self-reported measures of PA are prone to recall error and susceptible to social desirability biases [16]. Furthermore, the focus on PA has largely been on leisure-time activities, to the exclusion of other types of PA such as active transportation or other light-intensity activities (e.g., walking to school, playing catch, or golfing). Second, study designs are often based on retrospective recall asking participants to report their activity levels during high school in their first year following high school graduation [9, 11, 12]. These 8-to-12 month recalls are once again susceptible to recall errors, and the response accuracy would be greatly enhanced if studies began while students were still in high school.

In addition to the limitations in PA assessments, relatively few studies have investigated the factors influencing the changes in PA during the transition into emerging adulthood. Current evidence suggests that the lack of time and re-prioritization towards school work are salient barriers to PA for young adults entering postsecondary schools following high school graduation [17–19]. Findings from several other studies suggest that self-efficacy, and to a lesser degree behavioural intentions, are also factors significantly related to the participation of PA during this transitional period [14, 15, 20]. The body of literature, however, has tended to focus exclusively on the individual-level correlates of PA, to the exclusion of socio-environmental and contextual factors (e.g., social support, physical environment). Moreover, we are not aware of any studies that have examined changes in these determinants to explain the changes in PA.

The transition into early adulthood is considered by many as the first major transition that a person faces, representing a complex process that moves a youth away from parental support and dependency to taking definitive steps towards greater independence and autonomy [21, 22]. Given that intervention efforts targeting PA during this transition period are clearly required, our understanding of the factors related to behaviour change during this time can be strengthened in several ways. Firstly, there is a need for more studies with repeated assessments of PA cognitions to effectively understand the changes in motivations corresponding to changes in behavioural patterns. Additionally, given that much of the literature has almost exclusively focused on individual-level correlates of PA during this transition period [20, 23, 24], it will also be critical for studies to measure changes in social and environmental influences and understand the context in which changes in PA may be occurring. Therefore, the broader purpose of the MovingU Study is to conduct a cohort study to gain a much more comprehensive understanding of the behavioural patterns and the socio-ecological factors related to the changes in PA during the transition out of high school. Specifically, the objectives of this study will be to: (a) examine PA behavioural patterns during the acute transition out of high school, assessed through both objective and self-reported measures of activity; and (b) examine changes in psychosocial, socio-environmental, and contextual factors related to PA behaviours during this emerging adulthood period.

## Methods/Design

### Study design and participants

**Fig. 1:**
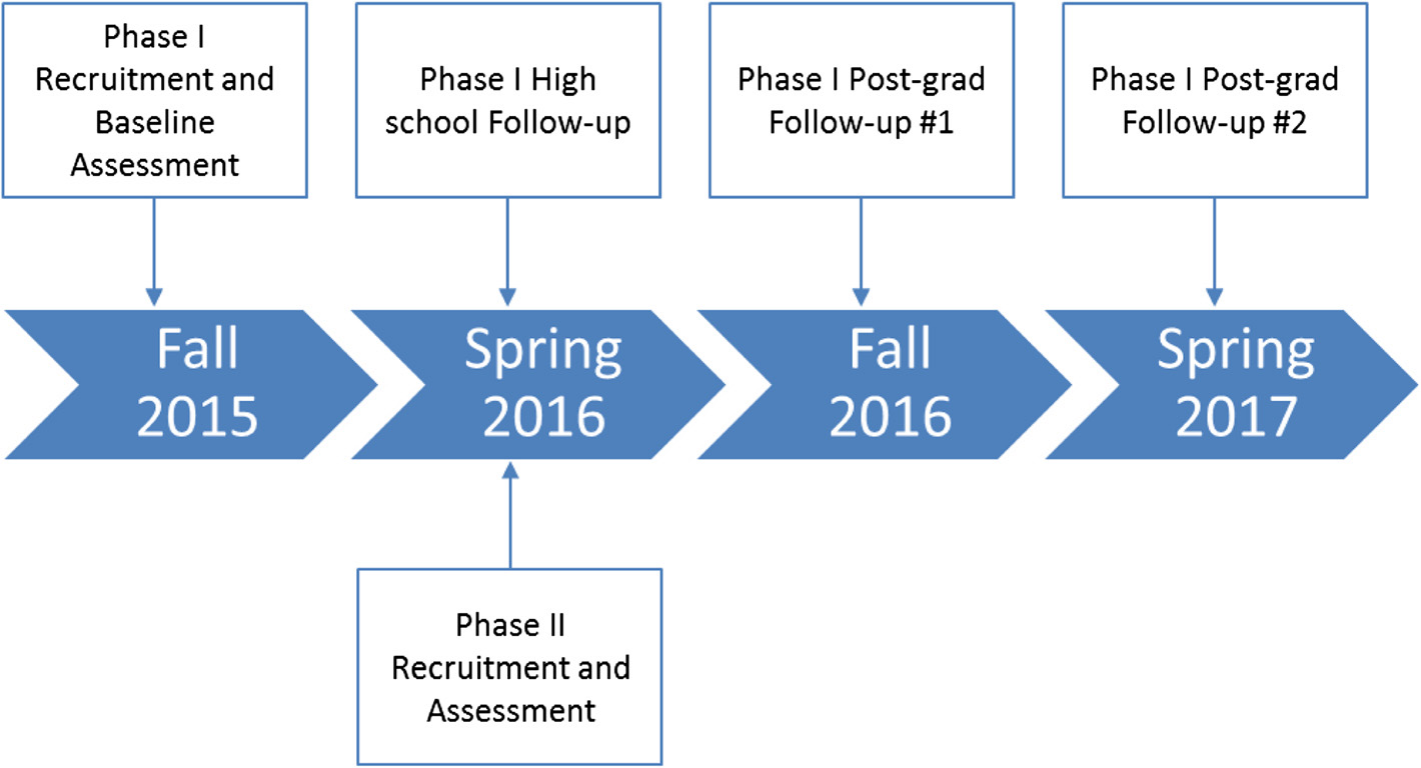
Timeline for MovingU Study

The MovingU Study is comprised of two phases capturing the broader emerging adulthood period. Phase I is a prospective cohort study that will follow approximately 120 impending high school graduates over the course of 18 months (Fig. 1). Participants will be initially recruited during the beginning of their grade 12 semester, with three additional follow-ups occurring approximately every 24-weeks. Using G*Power (version 3.1), we have determined that a minimum cohort of 78 participants will permit the detection of standardized effect size of 0.3 in the psychosocial predictors related to PA among young adults with 80 % power and a significance level set at 0.05. To account for the likelihood of a sizable attrition rate (20 %) during follow-ups, approximately 200 students will be recruited at baseline. During each of the data collection periods, students will be asked to complete a questionnaire inclusive of various psychosocial and socio-environmental measures, and be asked to wear the ActiGraph GT9X Link, a wrist-worn accelerometer for a period of seven days.

Phase II is conducting a cross-sectional study of recent high school graduates using a real-time data capture strategy called Ecological Momentary Assessment (EMA) (Fig. 1). This method involves intensive sampling, where participants will be asked to complete very brief questionnaires (approximately 1-to-2 min) seven times each day for five days using their smartphones. We will use a random sampling schedule, meaning participants will be prompted at random times within pre-determined timeframes throughout the day (e.g., 9 am-11 am, 11 am-1 pm… 9 pm-11 pm). An independent sample of approximately 120 students will be recruited for this phase of the MovingU study. In addition to being asked to complete the series of EMA prompts for the duration of five days, participants will be required to wear the ActiGraph GT9X Link. The Hamilton Integrated Research Ethics Board and the Halton District School Board have both provided formal approval for all study protocols.

### Recruitment strategy & study protocol

#### Phase I

**Fig. 2:**
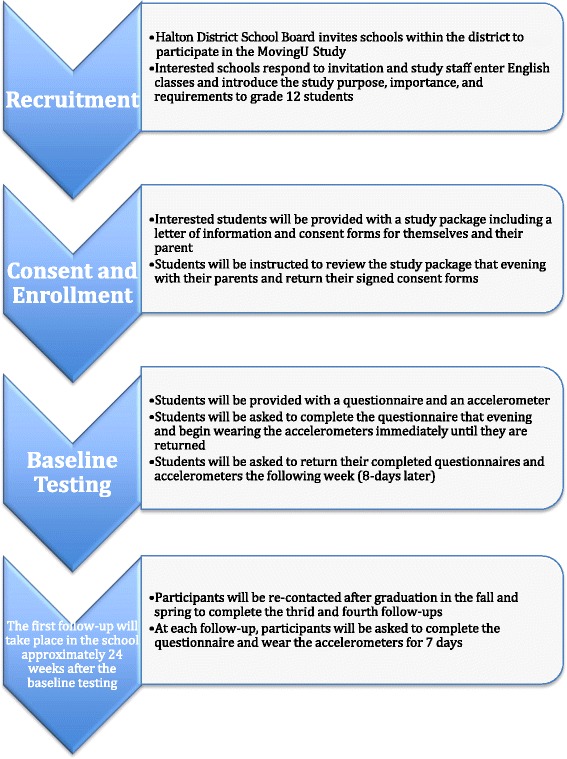
Recruitment Strategy and Study Protocol for Phase I

Our sample for phase I will be drawn from high schools within the Halton District School Board (HDSB) in Southern Ontario (Fig. 2). In an effort to obtain a relatively representative sample, we will specifically recruit students from grade 12 English classes. This is the only required subject during the final year of high school. All 18 high schools within the HDSB will be sent an initial invitation from HDSB to participate in the study. The first three or four schools that respond to the invitation will be included in the study in order to reach the targeted sample size, depending on the number of English classes at each school.

To recruit from the participating high schools, a trained research assistant will go into each of the English classes to provide a brief 5-min overview of the study. All students will be briefed on the study purpose, importance, and requirements, and asked if they would be interested and willing to participate. Students expressing interest in participating in the study will be provided with a study package: including a letter of information, consent forms to be completed by themselves as well as parental consent (permission for their child to take part in the study), a questionnaire, and an accelerometer with instructions for use. Students receiving the study package will be instructed to complete the consent forms and questionnaire that evening, and to begin wearing the accelerometer until they return all the study material the following week (8-days later). We will return to the high school approximately 24-weeks later to conduct the first follow-up assessment. Students will be required to complete a questionnaire and to wear the accelerometer for a period of seven full days. The latter two follow-ups will be conducted following the high school graduations of the participants. For each of these data collection periods, we will attempt to re-contact each participant, and if they wish to continue their participation in the study, they will be mailed a study package inclusive of the questionnaire, accelerometer, and a pre-paid envelope to send back the study material to the research team. Participants will be given a $25 Starbucks gift card as compensation for each of the data collection periods they take part in.

#### Phase II

Phase II of the study will take place at the student residence complexes at a large university, targeting first-year university students with an Android or iOS based smart-phone. Participants will be recruited over the course of two weeks and asked to participate by completing a questionnaire, wearing an accelerometer, and responding to multiple EMA prompts each day for five consecutive days. Recruitment materials (i.e., social media, flyers) will sent out ahead of time to all the students living in those residence buildings, with a brief explanation of the purpose of the study. Students interested in participating in the study will be asked to attend a brief presentation on one of two specified dates (each held on a Tuesday) for recruitment. The study will use a rolling recruitment strategy over the two weeks, with each data collection beginning on a Wednesday and ending on the Sunday.

During the briefing, students will be informed about the study purposes and requirements and will be asked to provide written consent. Subsequently, participants will be asked to complete an online questionnaire (same questionnaire as phase I) and to download the mEMA app (designed and developed by illumavu Inc. [25]) on their smartphone. Each person will be provided with a personalized code to enter into the app for the EMA prompts to be sent to their phones. A research assistant will also distribute accelerometers, and students will be instructed to wear it at all times, with the exception of showering and participation in water-based activities. As compensation for their time, students will be given a $10 Starbucks gift card for completion of the questionnaire and for wearing the accelerometer. Additionally, participants will be given $1 for each of the prompts that they completed, up to an additional $25 (in gift cards) over the five days.

### Measurements (Phase I & II)

#### Socio-demographic factors

Participants will be asked to complete a baseline demographic survey that includes items on age, gender, ethnicity, parental education, academic standing, and residential postal code (used as a proxy measure of household income). With the exception of academic standing, these socio-demographic factors will only be administered at baseline.

#### Objectively-assessed physical activity

Participants will be asked to wear an accelerometer (ActiGraph GT9X Link) on their non-dominant wrist for 5 (phase II) or 7 (phase I) days. This device will be set to collect data in 3 axes at a sampling rate of 30Hz. Participants will be instructed to wear the device for as much as possible while awake and sleeping, removing it only for prolonged water exposure. Wear time will be determined using the Troiano (2007) criteria, with any period ≥ 60 min of consecutive 0 counts flagged as non-wear. Days that the accelerometer was dropped off and picked up will be excluded from analyses. Physical activity data will only be analyzed for participants who meet the minimum wear time criteria (≥10 h on ≥3 days). Data will be analyzed using ActiLife Software (v.6.13.2) for daily total activity counts, average activity counts per minute, and daily steps.

#### Self-reported physical activity

Participants will be asked to complete the Modified Activity Questionnaire for Adolescents (MAQ-A) at each assessment. The MAQ-A is advantageous in that it allows respondents to identify the specific physical activities (i.e., organized sport, free-time activity, free play) that they have engaged in over the past 24-weeks, and the average frequency and duration of their participation [26]. Based on an established compendium of physical activities [27], each reported activity will be subsequently converted into estimated energy expenditures. The MAQ-A has been previously used in a population of adolescent students to measure PA and demonstrates adequate validity and reliability [26].

#### Psychosocial measures

Each assessment will have participants complete a battery of psychosocial and socio-environmental measures. The psychosocial and socio-environmental items are largely based on Bandura’s Social Cognitive Theory [28] and Health Action Process Approach (HAPA) model [29].

*Outcome Expectancy* will be assessed based on previous research examining the affective and instrumental components of outcome expectancies [30]. Participants will be asked to respond to a five-item measure asking “For me, engaging in at least 150 min of moderate-to-vigorous [MVPA] per week would…” [improve how I feel about myself; improve my health or decrease the risk of disease; help control my weight; feel less tense or stressed; increase my energy level]. Participants will indicate their agreement with each question using a 7-point Likert scale from 1 = *strongly disagree* to 7 = *strongly agree*.

*Task and Barrier self-efficacy* will each be measured using a single item asking about their belief in being physically active, consistent with McAuley and Mihalko’s [31] guide for assessing self-efficacy. The specific items ask participants “I am confident that I can be physically active (i.e., ≥ 150 min of MVPA each week)” and “I am confident that I can be physically active, even when I encounter barriers”. Participants will indicate their agreement with each question using a 7-point Likert scale from 1 = *strongly disagree* to 7 = *strongly agree*.

*Behavioural Intention* will be measured using a single item measure commonly used in the PA domain. Participants will be asked to rate the extent to which the following statement is likely: “I intend to do at least 150 min per week of moderate-to-vigorous physical activity”. Response options will range from 1 = *extremely unlikely*; 7 = *extremely likely*.

*Action control* items will assess participants’ awareness of their PA behaviours and their use of strategies for self-regulation. Based on previous work focused on action control [32], participants will be asked to respond to the degree to which each of the following statements are true: (1) I constantly monitor whether I engage in MVPA often enough; (2) Physical activity is often on my mind; (3) I really try to engage in PA of at least moderate-intensity regularly; (4) I try my best to meet my own standards for being physically active. Response options range from 1 = *definitely false* to 7 = *definitely true*.

*Action planning* will be assessed with five items that have been used in previous research examining the action phases within the broader motivational process [30]. Specifically, participants will indicate whether they generally make detailed plans regarding (a) where (e.g., at a fitness centre); (b) when (e.g., Monday’s after lunch); (c) how (take a bus to go climbing); and (d) with whom (e.g., my roomates) they will be engaging in MVPA with. Participants will respond on a 7-point Likert scale from 1 = *strongly disagree* to 7 = *strongly agree*.

#### Social support measure

The Multidimensional Scale of Perceived Social Support (MSPSS) will be used to assess social support [33]. Specifically, the 12-item measure includes three subscales, addressing a different source of support by either (a) family, (b) friends, and (c) significant others. For example, students are asked to what extent they agree with statements such as “I get the emotional help and support I need from my family”, “My friends really try and help me”, and “There is a special person in my life whom I can share my happiness and sadness”. Responses are measured on a 7-point Likert scale from 1 = *very strongly disagree* to 7 = *very strongly agree*. Previous research has demonstrated that the MSPSS has good internal and test-retest reliability as well as good construct validity [34].

#### Mental health measures

The Kessler Psychological Distress Scale 10 (K10) [35] and a modified version of the General Health Questionnaire-28 (GHQ-28) [36] will be used to assess mental health outcomes. The K10 is designed as a brief screening tool that assesses psychological distress in the general population using 10 items [35]. For example, students are asked to indicate how frequently they have felt depressed or lonely over the past month, and respondents indicate the degree to which they have felt this way on a 4-point scale (1 = *none of the time,* 2 = *some or little of the time,* 3 = *occasionally or moderate amount of time*, 4 = *all the time*). The GHQ-28 is another diagnostic screening tool that assessed four sub-domains of mental health, including social dysfunction, anxiety-insomnia, major depression, and somatic symptoms [36]. For example, questions asked participants: “Thinking of the last 2 months, have you felt capable of making decisions about things?” or “Thinking of the last 2 months, have you felt constantly under strain?” Each subscale includes 7 items, however, due to concerns over the sensitive nature of some items on the severe depression subscale, only 3 items will be used. Responses range from 1 = *less so than usual,* 2 = *same as usual,* 3 = *rather more than usual*, 4 = *much more than usual* [35, 36].

### Ecological momentary assessment measures (Phase II only)

As previously mentioned, participants in phase II will be required to complete a series of seven EMA prompts each day over the five-day study period. Given the intensive sampling strategy, very brief questionnaires were developed to reduce participant burden (i.e., surveys designed to take one-to-two minutes to complete). Furthermore, if an EMA prompt were to occur during an incompatible activity (e.g., in class, shower, driving), participants will be instructed to ignore it. If a survey prompt is not immediately answered, there will be two additional reminders sent every five minutes, each with a five-minute window to complete, after which the survey becomes inaccessible until the next prompt. Survey responses are manually uploaded by the participants, with the option of doing so intermittently or at the end of the study. The EMA questions will assess self-reported contextual information on current activity, physical location, type of social company, current affective and feeling states, as well as state motivation to be active and self-control.

#### Context-specific measures

Each prompt sent to participants will include questions to obtain contextual information on three domains: (a) what participants are currently doing, (b) where participants currently are, and (c) with whom they are currently accompanied by. These items have been used in previous research, and have been found to be valid measures [37, 38]. Specifically, the questions will ask: “What are you currently doing?”, “Where are you right now?”, and “Who are you currently with?”*,* each including a multiple choice of likely responses (e.g., studying/homework, watching television, or eating or drinking; at home in my residence, on-campus indoors, or off-campus; by myself, with friends, or with classmates, etc.). O*ther* response options will also be available to allow participants to further specify in text the what, where, and with whom participants were with when responding to the EMA prompts.

#### Affective and feeling state

Based on previous EMA research [39], we will include seven items that ask about particular affective and feeling states on an 5-point adjectival scale (i.e., 1 = *not at all*, 2 = *a little*, 3 = *moderately*, 4 = *quite a bit*, 5 = *extremely*); five of the seven items will be randomly presented at each of the survey prompts throughout the day. For example, the questions will ask “how happy do you feel right now?”, “how tense or anxious do you feel right now?”, and “how fatigued or tired do you feel right now?”

#### State physical activity motivation

Each prompt will also include measures of acute outcome expectancy, barrier self-efficacy, and intentions. For example, a specific question will ask “doing 10 + min of physical activity in the next few hours would help me feel less stressed”, “Can you do 10 + min of physical activity sometime within the next few hours, even if you get busy?” and “I intend to be physically active for 10 + min sometime within the next few hours”. Participants will respond on a 5-point scale to each of the six state motivations questions during each prompt, indicating their agreement with each item.

#### State self-control

Two items from the State Self-Control Capacity Scale (short version) [40] will be used for the study. Specifically, the questions ask participants: “If I were tempted by something right now, it would be very difficult to resist” and “I feel like my will power is gone”. Responses are on a 7-point Likert scale from 1 = *not true* to 7 = *very true.* In an effort to reduce participant burden, one of the two items will be randomly selected to be included in each of the survey prompts.

### Data analysis

#### Phase I

Our primary analytic plan consists of using mixed effects modeling with PA as our dependent measure. Given the inclusion of both objective and self-reported measures of PA, we will examine activity in several ways, including: estimated energy expenditure from accelerometers (e.g., total activity, MVPA); estimated energy expenditure from the MAQ-A; and participation in organized sport and leisure-time activities. Psychosocial and socio-environmental factors will be entered into the statistical model as time-varying independent variables, while socio-demographic factors (potential moderators) will be considered as time-invariant independent variables.

#### Phase II

Similar to previous EMA studies [39], patterns of unplanned missing data will first be evaluated to determine whether missingness is associated with any discernable characteristics. We will use two analytic strategies to address our specific research questions. First, to determine the contextual influence of acute PA, we will examine the EMA entries that were completed within 30 min prior to or after a recorded bout of 10 or more minutes of activity (as determined by time-stamped accelerometer data). Crosstab procedures will estimate the proportion of PA bouts occurring in different social (i.e., being with friends or family) and environmental (i.e., being indoors or outdoors) contexts. General estimating equations will be used to examine the associations between psychosocial and socio-demographic factors and PA occurring within different contexts (e.g., evening activities that are outside, or morning activities being indoors). Second, mixed effects location modeling will be used to examine the associations between intra-individual variability (i.e., degree of instability) in affective and feeling states and participation in PA. This technique will determine the relationship between variability in mood and feeling states as it relates to students’ overall PA behaviours.

## Discussion

Consistent with previous research [6–11], we expect that there will be dramatic decreases in PA behaviours during the transition out of high school. Understanding the factors that predict PA declines, however, are essential for developing effective interventions. Recognizing that PA is multifaceted, influenced by a complex interaction of transitional, social and economic issues, the MovingU Study is applying a socio-ecological approach to uncover salient factors influencing behaviour change and represents one of the first and most comprehensive studies to examine the changes in PA behaviours during the transition out of high school.

In addition to repeated assessments of individuals beginning at the start of their final year at high school for phase I of the study, we will be using both objective and self-reported measures of PA. This will allow us to effectively model patterns of PA behaviours, including the use of accelerometry data, which alone would make a substantial contribution to the literature. Repeated assessments of several known correlates of young adults’ PA will also enable us to examine the changes in theory-based psychosocial and socio-environmental factors; and subsequently to investigate how these changes relate to changing patterns of PA. Phase II of the study will examine how contextual environmental factors impact PA behaviours following high school graduation. Very little is known about how socio-environmental characteristics impact the experiences and behaviours related to PA in young adults, and this study will use an intensive data gathering strategy to better understand how their momentary states impact PA in an ecological manner. Collectively, the information will be critical in developing effective strategies to target PA both at high school and following high school graduation, and attenuate such drastic declines in PA behaviours.

## Abbreviations

EMA, ecological momentary assessment; GHQ-28, General Health Questionniare-28; HDSB, Halton District School Board; K10, Kessler Psychological Distress Scale 10; MAQ-A, Modified Activity Questionnaire for Adolescents; mEMA, mobile ecological momentary assessment application; MSPSS, Multidimensional Scale of Perceived Social Support; MVPA, moderate-to-vigorous physical activity; SPA, sport and physical activity

## References

[1] Colley RC, Garriguet D, Janssen I, Craig CL, Clarke J, Tremblay MS (2011). Physical activity of Canadian adults: accelerometer results from the 2007 to 2009 Canadian Health Measures Survey. Health Rep.

[2] Dishman RK, Washburn RD, Heath GW (2004). Physical Activity Epidemiology.

[3] Kohl HW, Craig CL, Lambert EV, Inoue S, Alkandari JR, Leetongin G (2012). The pandemic of physical inactivity: global action for public health. Lancet.

[4] Humphreys BR, McLeod L, Ruseski JE (2014). Physical activity and health outcomes: evidence from Canada. Health Econ.

[5] Katzmarzyk PT, Janssen I (2004). The economic costs associated with physical inactivity and obesity in Canada: an update. Can J Appl Physiol.

[6] Dumith SC, Gigante DP, Domingues MR, Kohl HW (2011). Physical activity change during adolescence: a systematic review and a pooled analysis. Int J Epidemiol.

[7] Gordon-Larsen P, Nelson MC, Popkin BM (2004). Longitudinal physical activity and sedentary behavior trends: adolescence to adulthood. Am J Prev Med.

[8] White P, McTeer W (2012). Socioeconomic status and sport participation at different developmental stages during childhood and youth: Multivariate analyses using Canadian national survey data. Sociol Sport J.

[9] Bray SR, Born HA (2004). Transition to university and vigorous physical activity: implications for health and psychological well-being. J Am Coll Heal.

[10] Kwan MY, Cairney J, Faulkner GE, Pullenayegum EE (2012). Physical activity and other health-risk behaviors during the transition into early adulthood: a longitudinal cohort study. Am J Prev Med.

[11] Wengreen HJ, Moncur C (2009). Change in diet, physical activity, and body weight among young-adults during the transition from high school to college. Nutr J.

[12] Edmonds MJ, Ferreira KJ, Nikiforuk EA, Finnie AK, Leavey SH, Duncan AM (2008). Body weight and percent body fat increase during the transition from high school to university in females. J Am Diet Assoc.

[13] Van Dyck D, De Bourdeaudhuij I, Deliens T, Deforche B (2015). Can changes in psychosocial factors and residency explain the decrease in physical activity during the transition from high school to college or university?. Int J Behav Med.

[14] Kwan MYW, Bray SR, Martin Ginis KA (2009). Predicting physical activity of first-year university students: An application of the theory of planned behavior. J Am Coll Heal.

[15] Bray SR (2007). Self-Efficacy for coping with barriers helps students stay physically active during transition to their first year at a university. Res Q Exerc Sport.

[16] Welk GJ, Corbin CB, Dale D (2000). Measurement issues in the assessment of physical activity in children. Res Q Exerc Sport.

[17] Kwan MY, Faulkner GE (2011). Perceptions and barriers to physical activity during the transition to university. Am J Health Stud.

[18] Gyurcsik NC, Spink KS, Bray SR, Chad K, Kwan M (2006). An ecologically based examination of barriers to physical activity in students from grade seven through first-year university. J Adolesc Health.

[19] Gómez-López M, Gallegos AG, Extremera AB (2010). Perceived barriers by university students in the practice of physical activities. J Sports Sci Med.

[20] Keating XD, Guan J, Piñero JC, Bridges DM (2005). A meta-analysis of college students’ physical activity behaviors. J Am Coll Heal.

[21] Brooks JH, DuBois DL (1995). Individual and environmental predictors of adjustment during the first year of college. J Coll Stud Dev.

[22] Jekielek S, Brown B. The transition to adulthood: Characteristics of young adults ages 18 to 24 in America. Baltimore, MD: Annie E. Casey Foundation, Population Reference Bureau, and Child Trends; 2005.

[23] Sallis JF, Prochaska JJ, Taylor (2000). A review of correlates of physical activity of children and adolescents. Med Sci Sports Exerc.

[24] Buckworth J, Nigg C (2004). Physical activity, exercise, and sedentary behavior in college students. J Am Coll Heal.

[25] mEMA by ilumivu [Internet]. [cited 2016 Jun 10]. Available from: https://ilumivu.wordpress.com/. Accessed 15 Apr 2015.

[26] Aaron DJ, Kriska AM, Dearwater SR, Cauley JA, Metz KF, LaPorte RE (1995). Reproducibility and validity of an epidemiologic questionnaire to assess past year physical activity in adolescents. Am J Epidemiol.

[27] Ainsworth BE, Haskell WL, Whitt MC, Irwin ML, Swartz AM, Strath SJ (2000). Compendium of physical activities: an update of activity codes and MET intensities. Med Sci Sports Exerc.

[28] Bandura A. Social foundations of thought and action: A social cognitive theory. Englewood Cliffs, NJ: Prentice-Hall, Inc; 1986.

[29] Schwarzer R, Schwarzer R (1992). Self-efficacy in the adoption and maintenance of health behaviors: theoretical approaches and a new model. Self-Efficacy: Thought Control of Action.

[30] Arbour-Nicitopoulos KP, Duncan M, Remington G, Cairney J, Faulkner GE (2014). Development and reliability testing of a health action process approach inventory for physical activity participation among individuals with schizophrenia. Front Psychiatry.

[31] McAuley E, Mihalko SL. Measuring exercise-related self-efficacy. Adv Sport Exerc Psychol Meas. 1998;371–90.

[32] Sniehotta FF, Scholz U, Schwarzer R (2005). Bridging the intention–behaviour gap: Planning, self-efficacy, and action control in the adoption and maintenance of physical exercise. Psychol Health.

[33] Zimet GD, Dahlem NW, Zimet SG, Farley GK (1988). The multidimensional scale of perceived social support. J Pers Assess.

[34] Zimet GD, Powell SS, Farley GK, Werkman S, Berkoff KA (1990). Psychometric characteristics of the multidimensional scale of perceived social support. J Pers Assess.

[35] Kessler RC, Andrews G, Colpe LJ, Hiripi E, Mroczek DK, Normand S-L (2002). Short screening scales to monitor population prevalences and trends in non-specific psychological distress. Psychol Med.

[36] Goldberg D, Williams P (1988). A user’s guide to the General Health Questionnaire.

[37] Dunton GF, Intille SS, Wolch J, Pentz MA (2012). Children’s perceptions of physical activity environments captured through ecological momentary assessment: a validation study. Prev Med.

[38] Liao Y, Intille SS, Dunton GF (2015). Using Ecological Momentary Assessment to understand where and with whom adults’ physical and sedentary activity occur. Int J Behav Med.

[39] Dunton GF, Huh J, Leventhal AM, Riggs N, Hedeker D, Spruijt-Metz D (2014). Momentary assessment of affect, physical feeling states, and physical activity in children. Health Psychol.

[40] Ciarocco NJ, Twenge JM, Muraven M, Tice DM (2004). Measuring state self-control: reliability, validity, and correlations with physical and psychological stress.

